# Metric partnerships: global burden of disease estimates within the World Bank, the World Health Organisation and the Institute for Health Metrics and Evaluation

**DOI:** 10.12688/wellcomeopenres.15011.2

**Published:** 2020-01-29

**Authors:** Marlee Tichenor, Devi Sridhar

**Affiliations:** 1 Global Health Governance Programme, University of Edinburgh, Edinburgh, UK

**Keywords:** global burden of disease, estimation, World Health Organisation, World Bank, Institute for Health Metrics and Evaluation

## Abstract

The global burden of disease study—which has been affiliated with the World Bank and the World Health Organisation (WHO) and is now housed in the Institute for Health Metrics and Evaluation (IHME)—has become a very important tool to global health governance since it was first published in the 1993 World Development Report. In this article, based on literature review of primary and secondary sources as well as field notes from public events, we present first a summary of the origins and evolution of the GBD over the past 25 years. We then analyse two illustrative examples of estimates and the ways in which they gloss over the assumptions and knowledge gaps in their production, highlighting the importance of historical context by country and by disease in the quality of health data. Finally, we delve into the question of the end users of these estimates and the tensions that lie at the heart of producing estimates of local, national, and global burdens of disease. These tensions bring to light the different institutional ethics and motivations of IHME, WHO, and the World Bank, and they draw our attention to the importance of estimate methodologies in representing problems and their solutions in global health. With the rise in the investment in and the power of global health estimates, the question of representing global health problems becomes ever more entangled in decisions made about how to adjust reported numbers and to evolving statistical science. Ultimately, more work needs to be done to create evidence that is relevant and meaningful on country and district levels, which means shifting resources and support for quantitative—and qualitative—data production, analysis, and synthesis to countries that are the targeted beneficiaries of such global health estimates.

## Introduction

At the 71 ^st^ World Health Assembly in Geneva in May 2018, a political alliance was struck between Chris Murray, Director of the Institute for Health Metrics and Evaluation (IHME), and Tedros Adhanom Ghebreyesus, Director-General of the World Health Organisation (WHO). IHME and WHO signed a Memorandum of Understanding (MOU), attempting to resolve what Lancet editor-in-chief Richard Horton called “a Cold War” of methodological, institutional, and philosophical tensions that occasionally erupted between the two organisations since IHME was established in 2007 ^1^. Since IHME started putting out the results of its Global Burden of Disease (GBD) study in 2012 ^2, 3^, there have been controversial differences between IHME’s estimates of disease burden and those of WHO ^4, 5^, particularly around malaria ^6^, tuberculosis ^7^, causes of child death ^8, 9^, and maternal mortality ^10^.

The explicit agreement by IHME and WHO to produce a single GBD study—“a series of capstone papers summarizing high-level findings would be published in *The Lancet* ^11^” before being used in official WHO documents—raises important questions about what an alliance of global burden of disease estimates means for global health governance and health policy in the global South. In official language, these estimates’ producers have always meant them to go hand in hand with the development of better data collection and vital registration systems in countries with limited data collection infrastructure ^12, 13^. In the 2018 MOU itself, in fact, both WHO and IHME assert that “estimates are no replacement for data from strong surveillance systems ^11^”. In reality, producing burden of disease estimates has always required WHO to reconcile the differences between its own disease estimates and country reported numbers. With this new alliance, the organisation will also have to reconcile estimates and methodologies from IHME with its own estimates and country reported numbers, as well as the two very different philosophical perspectives of the two institutions. These acts of reconciliation are mirrored by the attempted alliance between the World Bank and IHME to produce the Human Capital Index and the methodological tensions revealed between the two organisations with regards to the use of global disease burden estimates ^14^.

The practice of estimation in health development is by no means new. Early examples of the use of mathematical models based on assumptions include Daniel Bernoulli’s 1766 predictions of smallpox morbidity and mortality rates, should the English government not take on inoculation practices ^15, 16^. However, as has been remarked by many scholars ^17^, the amount of money and labour that went into estimation-production radically increased in the era of the Millennium Development Goals and is expected to grow in the era of measuring the progress of the much more complicated Sustainable Development Goals. This includes IHME’s production of the SDG-index, which was created particularly with the aim to assess the SDGs’ measurability ^18^.

In this article, we present first a summary of the origins and evolution of the GBD, which has been the object of much scrutiny ^19^, over the past 25 years. Then, we analyse two illustrative examples of GBD estimates and the ways in which they gloss over the assumptions and knowledge gaps in their production, highlighting the importance of the historical context by country and by disease in the quality of health data. We show how these estimates gloss over their assumptions and knowledge gaps, attempting to quantify context and data quality. Finally, we delve into the question of the end users of these estimates. Who uses these estimates and who does not, and what tensions lie at the heart of producing estimates of local, national, and global burdens of disease? These tensions bring to light the different institutional ethics and motivations of IHME, WHO, and the World Bank, and draw our attention to how the production of data and estimates is key to representing problems and their solutions in global health. With the rise in investment and power of global health estimates, the question of representing global health problems becomes even more entangled in decisions made about how to adjust reported numbers and statistical equations. The tensions at the heart of the GBD study are of utmost importance because those who represent global health problems are also those who determine how money and influence flow to address them.

## Methods

This analysis paper relies on two data sources. First, we used published articles to construct a timeline of the history and methodological conflicts of the global burden of disease study. Second, we analysed popular media representations of partnerships between WHO, IHME, and the World Bank, as well as field notes taken by M.T. at three public events at IHME and the World Bank. These three events were IHME’s 20th Global Burden of Disease Anniversary event in September 2017, IHME’s Annual Board Meeting in June 2018, and Sir George Alleyne’s lecture on human capital at the World Bank headquarters in July 2018. The paper does not attempt to give a comprehensive history of the GBD study—which over the course of its twenty-five-year history has included a very large cast of contributors, including those who have generously provided feedback on the first version of this article, and which has been the topic of extensive debate—but instead provides an introductory history of the development of the study and key moments in this debate in order to discuss the implications of two recent attempted and achieved partnerships between IHME, the World Bank and WHO (the Human Capital Project with the World Bank, and the 2018 MOU with WHO to create a single set of global health estimates).

## Key historical moments and tensions at the heart of the Global Burden of Disease study

The GBD and the disability-adjusted life year (DALY) have been subject to detailed scrutiny since their inception in the early 1990s. The DALY and the GBD first came onto the world scene out of a partnership between Chris Murray, Alan Lopez, and Dean Jamison ^a^, with the explicit institutional support of Harvard University, WHO, and the World Bank ^20^. The Bank requested “a comparative, comprehensive, and detailed study of health loss worldwide to provide the basis for objective assessments about the probable benefits of applying packages of interventions ^21^,” as part of the Bank’s increasing influence and financial investment in international health development in the 1980s and early 1990s ^22^. The team’s work culminated in the publication of the 1993 World Development Report (WDR), *Investing in Health* ^23^, which synthesized decades of work in health economics into the new health metric, the DALY, to provide punchy, useable language for justifying public and private, national and international investment in health. Murray and Lopez themselves define the study as a “systematic scientific effort to quantify the comparative magnitude of health loss from diseases, injuries, and risks by age, sex, and population over time ^21^”.

The three explicitly stated aims of the GBD were “(i) to decouple epidemiological assessment of the magnitude of health problems from advocacy by interest groups of particular health policies or interventions; (ii) to include in international health policy debates information on non-fatal health outcomes along with information on mortality; and (iii) to undertake the quantification of health problems in units that can also be used in economic appraisal ^24^”. The study’s architects were motivated by providing estimates on morbidity rates, as well as mortality rates, on diseases and conditions from a neutral, objective perspective. These estimates were built to be plugged into economic appraisal calculations to give international health organisations and governments guidance for prioritising health interventions based on economic reasoning. Until that point, assessments of global disease morbidity and mortality rates had been the responsibility of different programmes at WHO that focused only on specific diseases and interested largely with mortality rates, with estimates that leant heavily toward overestimation ^25^. The DALY, as a metric which quantified morbidity over time, was introduced as a means of drawing global attention to diseases and injuries that burdened populations but did not always result in death.

For all future GBD studies, 1990 served as the benchmark year, and over the course of the GBD’s life, mortality and morbidity estimates for that year were consistently reworked based on new data inputs and new estimation procedures. The next big moment for the GBD was 1997, when Richard Horton at *The Lancet* published the first peer-reviewed series of articles based on the research that was at the heart of the 1993 WDR, which Murray and Lopez call the “first complete revision of the GBD 1990 study ^21^.” The publication of these four articles ^12, 26– 28^ in *The Lancet* was so foundational to the genesis of the GBD that it served as the starting point for IHME’s 20 ^th^ anniversary celebration of the event in Seattle in 2017. Still using the year 1990, these articles addressed a few of the major concerns that scholars had identified in the original study published in 1993, including the criticism of the way the study weighted DALYs by age ^29^. The DALY quantifies healthy time lost to illness or death against a standardised average healthy life expectation, by combining years of life lost to death (YLL) and years of life lost to disability (YLD). The healthy life expectancy (HALE) is estimated for every population represented in the study—determined by location, sex, and year—and it is the reference against which YLLs and YLDs are subtracted to determine DALYs for each population.

In each cycle of the GBD since, its architects have carefully reassessed the nature of the disability weights used to condition DALYs, among other changes over the decades ( Table 1). As authors at WHO assert is the case in their description of the evolution of the GBD, determining disability weights has proven to be one of the most contentious issues of producing global health estimates ^30^. Producing disability weights requires an agreement on how to “define, measure and numerically value time lived in non-fatal health states”. At the heart of determining this numerical value are debates about “societal” vs. “individual” judgements on the severity of certain losses of health over others, as well as whether to privilege health professionals’ perspective over the general population’s ^31^. In an attempt to address these conflicting perspectives on valuing health, the GBD and GHE since 2010 have based their disability weights on a “comprehensive re-estimation of disability weights” based on surveys from the “general population ^32^”.

**Table 1.** Key GBD studies from 1990 to the present. These are the official studies indicated as such by Chris Murray and Alan Lopez in their description of the evolution of the GBD ^33^. Key articles and methodological changes are described here, but they are by no means exhaustive.

GBD Study Year(s)	Years Included	Publication Year	Key Publications	Key Authors	Institutional Support	Disease/ Injury Categories (Morbidity)	Notable Changes to the Study
**1990**	1990	1993	*World Development* *Report: Investing in* *Health ^i^*	Murray, Lopez, Jamison	World Bank, WHO, Harvard University	109	
	1990	1997	*The Lancet*	Murray, Lopez	WHO, World Bank, Edna McConnell Clark Foundation, Rockefeller Foundation	107; 483 sequelae	•Eliminated age weighting
**1999–2004**	1999–2004	1999–2002	*World Health* *Reports ^I, ii, iii, iv^*	Murray, Lopez, Mathers, Ma Fat	WHO		•New methodologies for finding mortality ^ii^
		2004	*The Global Burden* *of Disease: 2004* *Update*	Mathers, Ma Fat	WHO		•Incorporated cause-specific and multicause models – developed by the WHO Child Health Epidemiology Reference Group (CHERG) – for causes of child deaths under five years of age and for neonatal deaths
**2010**	1990, 2005, 2010	2012	*The Lancet* ^iii^	Murray, Lopez, *et al*.	IHME; The Gates Foundation	291; 1160 sequelae	•For the first time, quantified uncertainty for each estimation to provide a means for the user to judge its strength
**2013**	1990–2013	2014, 2015	*The Lancet* ^iv^	Murray, Lopez, *et al*.	IHME; The Gates Foundation	306; 2337 sequelae	•First of annual GBD updates
**2015**	1990–2015	2016	*The Lancet* ^v^	Murray, Lopez, *et al*.	IHME; The Gates Foundation	328; 2982 sequelae	•Introduction of Socio-demographic Index (SDI) based on measures of income per capita, average years of schooling, and total fertility rate
**2016**	1990–2016	2017	*The Lancet* ^vi^	Murray, Lopez, *et al*.	IHME; The Gates Foundation	333; 2982 sequelae	•Introduction of measuring progress of health-related Sustainable Development Goals •Added attention to subnational data
**2017**	1990–2017	2018	*The Lancet* ^vii^	Murray, Lopez, *et al*.	IHME; The Gates Foundation	354; 3484 sequelae	•Added “2842 collaborator-provided data sources and incorporating new clinical data representing an additional 149 million admissions and 3.7 billion outpatient visits for use in GBD modelling,” resulting “in a total of 68, 781 sources being used in the estimation process”

These are the GBD studies that Chris Murray and Alan Lopez indicate are the official ones in their own description of the evolution of the GBD ^viii^. Key articles and methodological changes are cited here, but they are by no means exhaustive.

^i^World Bank: World Development Report: Investing in Health. World Development Indicators. Oxford: Oxford Univ. Press. 1993.

^i^WHO: The World Health Report 1999: Making a Difference. Geneva: WHO. 1999.

^ii^WHO: The World Health Report 2000: Health Systems: Improving Performance. Geneva: WHO. 2000.

^iii^WHO: World Health Report 2001: Mental Health: New Understanding, New Hope. Geneva: WHO. 2002.

^iv^WHO: The World Health Report 2002: Reducing Risks, Promoting Healthy Life. Geneva: WHO. 2003.

^ii^Lopez AD, Ahmad OB, Guillot M, *et al.:* World mortality in 2000: life tables for 191 countries. Geneva: World Health Organization. 2002.

^iii^Murray CJL, Vos T, Lozano R, Naghavi M, Flaxman AD, Michaud C, Ezzati M, *et al.:* Disability-Adjusted Life Years (DALYs) for 291 Diseases and Injuries in 21 Regions, 1990–2010: A Systematic Analysis for the Global Burden of Disease Study 2010. *Lancet.* 2012. 380 (9859): 2197–2223.

^iv^Murray CJL, Barber RM, Foreman KJ, Ozgoren AA, Abd-Allah F, Abera SF, Aboyans V, *et al.*: Global, Regional, and National Disability-Adjusted Life Years (DALYs) for 306 Diseases and Injuries and Healthy Life Expectancy (HALE) for 188 Countries, 1990–2013: Quantifying the Epidemiological Transition. *Lancet.* 2015. 386 (10009): 2145–2191.

^v^Kassebaum NJ, Arora M, Barber RM, Bhutta ZA, Brown J, Carter A, Casey DC, *et al.*: Global, Regional, and National Disability-Adjusted Life-Years (DALYs) for 315 Diseases and Injuries and Healthy Life Expectancy (HALE), 1990–2015: A Systematic Analysis for the Global Burden of Disease Study 2015. *Lancet.* 2016. 388 (10053): 1603–58.

^vi^Hay SI, Abajobir AA, Abate KH, Abbafati C, Abbas KM, Abd-Allah F, Abdulkader RS, *et al.*: Global, Regional, and National Disability-Adjusted Life-Years (DALYs) for 333 Diseases and Injuries and Healthy Life Expectancy (HALE) for 195 Countries and Territories, 1990–2016: A Systematic Analysis for the Global Burden of Disease Study 2016. *Lancet.* 2017. 390 (10100): 1260–1344.

^vii^James SL, Abate D, Abate KH, Abay SM, Abbafati C, Abbasi N, Abbastabar H, *et al.*: Global, Regional, and National Incidence, Prevalence, and Years Lived with Disability for 354 Diseases and Injuries for 195 Countries and Territories, 1990–2017: A Systematic Analysis for the Global Burden of Disease Study 2017. *Lancet.* 2018. 392 (10159): 1789–1858.

^viii^Murray CJL, Lopez AD. Measuring Global Health: Motivation and Evolution of the Global Burden of Disease Study. *Lancet.* 2017. 390 (10100): 1460–64.

By 2000, Chris Murray had joined Alan Lopez at WHO, and they published their national health systems ranking using the GBD and the DALY, with the explicit institutional support of WHO. They had the particular support of Gro Harlem Brundtland and Julio Frenk, who were, respectively, Director-General of WHO and executive director of the Evidence and Information for Policy cluster at the time. This resulted in the publication of the 2000 World Health Report, *Health Systems: Improving Performance*. This exercise in explicitly naming and shaming countries for the performance of their health systems was highly controversial, as those countries that scored lower expressed their displeasure to WHO and as researchers and popular media pointed alternatively to WHO’s “underlying pro-market ideology ^34, 35^” and its “Marxist stance ^35, 36^”. However, as public health scholar Martin McKee argued, the 2000 WHR did in fact “place [the] assessment of health system performance firmly on the political and research agendas ^35^”. For the years 1999–2002 and 2004, WHO produced multiple GBD updates and published the estimates largely in the intervening World Health Reports ^37– 40^. These were borne out of the Global Programme on Evidence for Health Policy within WHO, part of the Evidence and Information for Policy cluster of which Murray became executive director when Frenk left to become the Mexican Minister of Health in the summer of 2000.

When Brundtland resigned from WHO in 2003, the GBD lost its most powerful supporter in the organisation, which led to the split between GBD studies, marked by Murray’s departure from WHO for Harvard University in 2003. In 2007, the Bill and Melinda Gates Foundation (BMGF), along with the University of Washington, funded Murray and Lopez to create an independent, scientific institution to produce estimates of global disease burden ^41^. Part of Murray and Lopez’s vision was to separate the production of estimates from the politics of vertical approaches and disease advocacy at WHO ^25^. IHME’s founding board members included Brundtland, Frenk, and Tedros. Given their large global health portfolio, BMGF also had a clear interest in ensuring timely, independent, and robust production of burden of diseases estimates, and IHME became their guide to do this. IHME lists impartiality as one of its five core principles: “For health evidence to be useful, it also must be credible, generated by a scientific process unimpeded by political, financial, or other types of interference. IHME was created to fill a gap in global health: to separate the measurement and evaluation of health policies and programs from the process of creating, implementing, and advocating for policies and programs ^42^”.

In 2012, the group published their first GBD study in *The Lancet*, covering the years 1990, 2005, and 2010, calling it the GBD 2010 ^43^. As we will touch on more extensively in the next section, this study caused a stir in the global health community, as some crucial estimates, like those of malaria morbidity and maternal mortality, diverged heavily from those put out by WHO and its UN agency partners for the same year ^44^. In 2015, IHME published the revamped GBD 2013 in *The Lancet*, including estimates for all of the years between 1990–2013, and since 2016, they have published complete annual reassessments of these years’ estimates, adding new assessments in each cycle. IHME’s GBD 2017 was published in *The Lancet* in November 2018.

The DALY, the metric at the heart of the GBD, was introduced explicitly as a means to draw global attention to diseases that do not only kill but also disable. By quantifying the loss of *healthy* years, the DALY was meant to unveil suffering, like lower back pain or depression, that weighs heavy on the world. Some scholars have argued that the metric, so ready to be used for cost-benefit analysis, and its proliferation more fundamentally have contributed to an intensification of economic rationality in and the neoliberalisation of global health thinking ^45, 46^. Medical anthropologist Vincanne Adams argues that “the DALY provides an economic measure of human productive value by calculating loss of productivity due to disease or disability ^47^”. As a tool that was created for the World Bank’s new increased investment in global health development, the DALY has from the start tied economic values to human suffering and attempts to alleviate it ^48^. Explicitly meant to be useable for ministers of finance and drawing a particular American version of economics into the sphere of international health development, the DALY has led to the reign of health economics in global health governance, a recent shift which Sridhar calls the economic gaze ^49^.

The GBD in its current form is also the result of a particular institutional view on the world. Expanding on Peter Byass’ conceptual framework of global health estimates ^50^, we argue that there are actually four different institutional positions that frame global health metrics production. We would call these four institutional positions “traditional academic population health department”, “think tank”, “health-policy oriented multilateral”, and “economic-policy oriented multilateral”, the last three of which are exemplified by IHME, WHO, and the World Bank. We argue that IHME is not a traditional academic population health department governed by academic publishing logics, which Byass contrasts against the governing logics of UN agencies’ global health estimate production. Although there are aspects of academic scientific rigor that define IHME’s estimate production—including oversight by an Independent Advisory Committee—they are also governed by the demand for deliverables by BMGF and have privileged access to private industry’s data streams because of their ambiguous institutional nature. As multilateral organisations, WHO and the World Bank have contractual relationships with officials in the countries with which they work, and thus are responsible for producing statistics that are legible to country officials. With different mandates for the ultimate goal of such production of statistics—one which places wellbeing above all else and the other economic prosperity above all else—WHO and the World Bank nonetheless are both required to harmonise the interests of a wide range of national, international, and civil society actors, including in the process of producing contextualised evidence for disease burden, which IHME asserts is a form of ‘politicizing’ evidence ^b^. The GBD in its current form has also been born out of collaborations with both WHO and the World Bank, meaning that the current collaboration with WHO is only the latest of a larger history of interactions and compromises between these three frameworks of measuring health’s progress and value in the world.

### On the production of the global burden of disease

One of the key challenges in producing a complete picture of the world’s health has been the lack of adequate civil registration and vital statistics systems or health information systems in several countries and the need to use a limited dataset to estimate the global burden of disease ^43^. This has resulted in a proliferation of modelling as a tool for estimating the larger picture, according to the data available. Inherent in producing estimates of population health is the question of how comfortable estimate producers are with extrapolating robust-seeming estimates from little or no data. “Imputing” data, in statistics parlance and the global health context, means bridging over conceived gaps in available data in one country with estimates based on data that does exist in comparable countries, often defined as comparable in terms of levels of GDP and regional proximity. This allows IHME, for example, to have estimates of malaria morbidity in the Central African Republic, where disease surveillance work has been incomplete since civil wars broke out in 2012 ^51^. Estimates’ level of uncertainty is directly related to the presence of health data infrastructure, meaning that estimates are least robust for countries with weaker health systems, which are often those countries perceived as needing disease burden estimates the most. Since one of IHME’s fundamental principles is that “[too] often, no estimate of a problem is interpreted as an estimate of no problem ^21^”, the organisation is known to be much more comfortable with imputation than the global health organisations that often use its data such as the World Bank, WHO, and UNICEF.

 However, imputing is not exclusively the practice of IHME. Various WHO, World Bank, and multilateral partnership programmes also practice imputation and other forms of data correction. While it is widely used, disagreements lie in the degree to which various organisations are comfortable with using imputation. Imputation and data correction can lead to tension between various producers of global health estimates, as well as between estimate producers and country officials. When the estimates of global disease and injury burden are used by other organisations or in health policy, they do not make explicit the complex methodologies nor the underlying primary data from country level involved in their creation. Discrepancies between IHME and WHO (and other UN agencies) sets of estimates have the effect of both reminding users of the complexities behind them, while also creating confusion for health policy makers ^17^. Even without having to address the discrepancies between its estimates and IHME’s, WHO has had to reconcile its own official estimates and country-reported morbidity and mortality rates. In its production of global and national health estimates, WHO uses multiple sources of data, although not as many as IHME, including Demographic Household Surveys (DHS) and the World Health Survey, to round out data provided through administratively reported data in public health clinics, in order to address potential bias within such production systems. As a result, WHO estimates can often be quite different than those that health ministers and finance ministers gather from their own statistics offices, and when global health estimates diverge dramatically from nationally gathered numbers, health ministers can unsurprisingly mistrust them ^17^.

Furthermore, on the scale of global health estimates themselves, one of the most controversial discrepancies between IHME’s and UN agencies’ estimates, as well as with nationally reported numbers, are those of malaria mortality rates ^6^. In 2010, IHME reported 1,238,000 deaths due to malaria, and that 524,000 of those were amongst individuals five years or older ^2^. For the same year, WHO reported 655,000 deaths in total with approximately 91,700 of those amongst individuals five years or older ^52^. This is due partly to the problem of a lack of information and diagnostic capacity in many places where malaria is endemic, but it is also due to discrepancies in defining the presence of malaria and the causality of a death. Since decreasing malaria morbidity and mortality was an explicit part of the Millennium Development Goals and the global health fight against malaria is heavily funded, this discrepancy caused a tumult in the global health world, which had three years to go to meet the Goals ^53^. Both IHME and WHO have used estimations of parasite density produced by the Malaria Atlas Project in the process of determining malaria mortality ^54^, but IHME‘s estimates also take into account a different interpretation of verbal autopsy data for morbidity in adults, based on the category of “fever of unknown cause ^55^”, potentially influenced by the Gates Foundation‘s political investment in the eradication of the disease.

Another notable discrepancy between the two sets of estimates is that of maternal mortality rates (MMR) ^10, 56^. In 2010, four UN agencies—UNICEF, UNFPA, the World Bank, and WHO—produced MMR estimates for the time period of 1990–2008 ^57^, while IHME produced estimates for the time period of 1980–2008 ^58^. For the final year of the study, 2008, the difference between the two estimates was low: IHME estimated 342,900 maternal deaths compared with the UN agencies’ estimate of 358,000. However, the two estimates differed markedly on their 1990 estimates, which changed the degree of global decrease in maternal deaths ^10^. As estimates are used by global health organisations and putatively for country-level health policy makers to measure success of certain kinds of interventions or approaches to health problems, differences in change certainly confound attempts to distinguish failing or successful health campaigns.

The fact of the matter is that the production of these estimates is conditioned by many levels of assumption, and they can result in numbers that are unrecognizable at the local level. In anthropologist-physician Clare Wendland’s analysis of maternal mortality ratio production and maternal care in a hospital in Malawi, she was confronted by health workers who expressed shock at the estimates produced far away that were being used to define MMR in the country: “They are saying we will meet the Millennium Development Goals. But I can’t believe it. If it’s that low, why are we still seeing this [much public mourning] every day? ^59^” Additionally, the availability and quality of local empirical data that serves as a starting point for these estimates are often determined by global health organisations’ priorities. Data collection systems may be thrown up around certain issues in conditions of precarity or where no problem was perceived before, such as cholera rates in post-disaster Haiti, malaria and HIV rates that were collected despite data retention strikes in Senegal ^60^, and Zika across the Americas in the wake of it being defined an epidemic by WHO ^61, 62^. What data are produced is defined by political and societal priorities and what types of data collection systems are funded by donors. This problem on the level of collecting empirical data becomes part of the larger political entanglements of the global health estimates they are used to produce.

### Who uses these estimates, and how?

The significant investment in disease burden estimates raises the question of who uses these estimates and how they are consumed. Tracking the use of its studies has proven difficult for IHME itself, which relies on following citation data, tracking the use of its data visualization tools, feedback from its collaborative network, and overseeing awards like its Roux Prize, to determine who and which agencies have used its data. From these sources, we can see that their data is used by other academic researchers (i.e., a high number of citations), global health organisations (i.e., the use of GBD estimates in policy reports), and, to a lesser degree, ministers of health and local politicians (i.e., the use of GBD estimates in national and subnational health policy). Organisations like WHO and the World Bank have at times used IHME’s data and at other times produced their own. The most direct consumer of this data is the Gates Foundation itself, the largest funder of IHME, which has mandated that the group produce a yearly revamp of their GBD study ^63^. The justification given is that BMGF uses IHME data to inform its investment portfolio. Since the organisation does not make its funding justification public, it is unclear how much GBD data is used regularly within BMGF to inform its funding portfolio.

With respect to WHO, the 2018 IHME-WHO Memorandum of Understanding is the second of its kind, the first having been signed in 2015. According to Boerma and Mathers, the first memorandum was signed “to encourage collaboration on country capacity strengthening, data sharing, and interaction on methods, tools, and actual global health estimates ^64^”. Part of this collaboration was the production of the Guidelines for Accurate and Transparent Health Estimates Reporting (GATHER), which was a response to a call by WHO in 2013 for consensus and better guidance for reporting and interpreting health data ^65^. When Dr. Tedros, who was on IHME’s founding board in 2007 as noted above, became WHO’s Director-General in 2016, he expressed his interest in reconciling the two systems of estimation. In the 2018 MOU, IHME and WHO carefully outline how the two organisations would collaborate on the General Programme of Work 2019–2023 (GPW 13), in policy dialogue and country capacity building, in publications, and specifically on the production of a single Global Burden of Disease study, the explanation of which occupies most of the document ^11^. The agreement will require IHME and UN agencies to confront the methodological tensions at the heart of their differing approaches. Whether it will result in a more careful approach to global health estimates, more explicitly communicating uncertainty and addressing ethical issues at the heart of the study are questions yet to be answered ^17^. The GBD alliance has the potential to create a global health data monopoly, extending the already extensive reach of the BMGF further into WHO in determining how global health problems are known and what kinds of approaches to health problems are viable.

In its continued relationship with the World Bank, IHME has been most recently tied to the Human Capital Project. In October 2017 at Columbia University, World Bank President Jim Kim formally announced the Human Capital Project, after first presenting on it at the 20 ^th^ GBD Anniversary event at IHME. He explained how the World Bank would publish the next generation of the health systems ranking from the 2000 World Health Report in Fall 2018, in partnership with IHME. The Human Capital Index would measure the educational attainment, educational quality, and functional health levels of each country ^66^. In his 2017 presentation at IHME, he argued for the inclusion of estimates from IHME’s GBD study in the proxy for functional health levels of each country. The goal of this new index was to measure countries’ investments in the education and health of its own citizenry. The larger human capital debate, of which the development of the DALY is a part, is a theoretical argument that economists have put forward to argue that education, health, and other social services are not *expenditures* but *investments* in a country’s economy ^67^. As Flabbi and Gatti explain, when Gary Becker first introduced the idea that “investing in human capital is akin to investing in physical capital,” it was quite controversial ^68, 69^. Even when the 1993 World Development Report ^70^ was released, which leans heavily on the human capital argument, there was push back from mainstream economists, as the Senior Director of the Health, Nutrition, and Population team, Tim Evans, reminded the audience at a talk on human capital at the World Bank in July 2018.

However, the execution of the Bank’s Human Capital Index project has resulted instead in two separate methodologies of estimating human capital, one produced by IHME ^71^ and one by the World Bank ^14^. This is a reversal of Bank President Jim Kim’s earlier assertion about forging a partnership between the two organisations. At the July 2018 talk on human capital at the World Bank, a team member of the Bank’s Human Capital Index project was asked why they were not taking advantage of IHME data and the “visibility” of the GBD and DALYs in their calculation and ranking of countries’ investment and status of education and health. The team member argued that both the WHO and IHME versions of the GBD used higher levels of imputation than his team habitually used. He acknowledged that they did go first to the IHME data, but quickly realized that there were many parts of the estimates that were based off of scarce empirical data. For the Bank team, this was problematic for three reasons. First, imputing data meant losing the line of sight for ministers of finance, ministers of health, and ministers of education. World Bank staff would not be able to remind ministers of which study was used to produce them nor the history and context of the numbers referenced, and thus it would not be recognizable on the country level. Second, Bank staff would then lose the platform for advocating for better data collection and the importance of addressing the structural problems and inequities that exist beneath the data gaps. Finally, tracking progress on these indicators then becomes very complicated, as estimates change year to year also due to changes in estimation or statistical science in addition to changes in material conditions. As Boerma and his colleagues remind us, “Neither country policy makers nor the global development community are best served by a global flood of health estimates derived from complex models as investments in country data collection, analytical capacity, and use are lagging ^64^”.

This methodological conflict between IHME and the World Bank shines a light on one of the conflicts at the heart of the use of IHME estimates in the monitoring of progress on the Sustainable Development Goals. There is first the problem mentioned here by the World Bank representative, and highlighted by others ^72^, that with the complete revamping of the methodology of the GBD study every year, there will always be confusion over whether progress from one year to the next is the result of change in the outside world or change in estimation science. Secondly, and most importantly for understanding the significance of the 2018 IHME-WHO MOU, is the nature of the conflict of interest at the heart of the BMGF nearly fully financially supporting the production of the GBD while also being one of the largest financial supporters of WHO. Because of the model of production of the GBD—which lies between and outside academic and multilateral frameworks and is not open for country-level review—this means that BMGF maintains outsized control over the means of actually measuring the success of its own investments within the UN agency system ^c^.

Beyond those critiques, a central value of the GBD can be found precisely in its ability to compare countries and regions in the context of health development. In the 2018 IHME-WHO MOU, they argue that the “GBD’s utility is largely for comparisons across locations and over time ^11^”. It is precisely this demand for comparability that calls and allows for the standardization of health data and the filling of data gaps ^17, 33^. However, it is worth asking the question of why global comparability is so important to achieving global health goals. The argument is that this work of comparing promotes healthy competition between countries, spurring those who see themselves as lagging behind into action, as global health leader Sir George Alleyne put it at a talk he gave on the World Bank’s Human Capital Project in July 2018. Alleyne added that there is something inherently human about thinking about the world in the framework of hierarchies and that statistics have long contributed to how we determine rank in such hierarchies. It is this assumption about the nature of competition that has prompted projects like the Human Capital Project, of course, and also the 2000 World Health Systems ranking.

In the quantification and rationalization of uncertainty, in an attempt to eradicate it, do other forms of evidence become delegitimized that are important in the process of determining health policy priorities? What are the larger ramifications of practices of standardization, data correction, and imputation that are performed particularly with the goal of making local contexts readable from a satellite’s view of the world? We would argue that it is certainly up for debate whether humans are doomed to be dominated by the work of competition rather than collaboration, and that there is an explicit history to the active construction and reification through statistics of “scientific” conceptions of hierarchies that benefit those who are at the top ^73, 74^.

## Conclusion

Fundamentally, the production of the GBD and its centrality to global health governance raises the question of the larger effects of producing numerical assessments of disease burden “from a distance ^75^”. How data is collected and how estimates are produced actually shape how we understand global health problems and their potential solutions, and thus estimates should never be taken for granted. When health workers, health policy makers, or even patients do not recognize their experiences in these estimates, like the Malawian physician mentioned above, the usefulness of such estimates and the kinds of knowledge with which they must be accompanied should be assessed. These estimates carry with them particular assumptions about the nature of illness and economics that attempt to universalize experiences of suffering and its impacts on our lives. They are excellent advocacy tools, but unfortunately their power extends beyond merely highlighting a problem, as they also leverage assumed health interventions along with them. What happens far less frequently, with important exceptions ^33, 76^, is the transfer of skills necessary to produce these estimates on a much more local level and investment in vital registration systems and health information systems ^77, 78^.

When numbers are called upon to “speak for” the health needs and priorities of populations in the global South, we must also ask the question of who produces these numbers, their apparent apolitical neutrality, and the broader governance structures that allow for them to hold the power they do. This does not mean that these estimates should not be produced, as global burden of disease estimates are an important “first pass” of the health profiles of different countries. However, they cannot be proxies for suffering in and of themselves, and global health benefits greatly from the visibility of the scientific and methodological conflicts that are at the heart of tensions between the two sets of powerful health estimates produced by WHO and by IHME. Ultimately, more work needs to be done to create evidence that is relevant and meaningful on country and district levels, which means shifting resources and support for quantitative—and qualitative—data production, analysis, and synthesis to countries that are the targeted beneficiaries of such global health estimates.

## Data availability

The primary and secondary sources used are cited. M.T.’s field notes from the three public events are under restricted availability. See the American Anthropological Association’s 2003 “ Statement on the Confidentiality of Field Notes” for more information. However, highly redacted versions of them may be available for academic research purposes. In order to access these highly redacted field notes, please contact M.T. ( marlee.tichenor@ed.ac.uk), outlining why the field notes would be relevant for your research and how they will be used.

## Footnotes

^a^These three individuals are listed as key three among many others whose labour were essential to the process of producing and disseminating the global burden of disease study.

^b^We thank our reviewer Colin Matthews for emphasising this point.

^c^We thank our reviewers, Carla AbouZahr and Peter Byass, for emphasising the importance of this point.
