# Supplementary material for: Metric partnerships: global burden of disease estimates within the World Bank, the World Health Organisation and the Institute for Health Metrics and Evaluation
Source: Wellcome Open Res. 2020 Jan 29;4:35. Originally published 2019 Feb 18. [Version 2] doi: 10.12688/wellcomeopenres.15011.2 (PMC6406176; doi:10.12688/wellcomeopenres.15011.2)
Supplement: Peer review history

# Peer review: Reviewer response for version 2

**DOI:** 10.21956/wellcomeopenres.17166.r37735

**Published:** 2020-02-06

**Authors:** Carla AbouZahr

**Affiliations:** 1 Bloomberg Data for Health Initiative, Melbourne, Australia; 2 CAZ Consulting Sárl, Geneva, Switzerland

I think the authors have done an excellent job in responding to the comments and have nothing further to add.

I confirm that I have read this submission and believe that I have an appropriate level of expertise to confirm that it is of an acceptable scientific standard.

# Peer review: Reviewer response for version 1

**DOI:** 10.21956/wellcomeopenres.16376.r34900

**Published:** 2019-03-06

**Authors:** Ties Boerma

**Affiliations:** 1 Centre for Global Public Health (CGPH), University of Manitoba, Winnipeg, Canada

The Global Burden of Disease (GBD) has become a major instrument in global health and appears destined to become even more important, if only because of the massive investments by the Gates Foundation into the production of GBD estimates. This paper reviews the position of the GBD as a tool in global health in the light of a recent agreement between IHME and WHO to produce a single GBD study, focusing on possible positive and negative implications of such an agreement.

 The paper is a useful piece to stimulate debate. The methods used by the authors have limitations (review of literature review and field notes from three public events) and could have benefited from more extensive and systematic research on the subject. This would have led to a more comprehensive understanding of the history of GBD and relevant concurrent developments in the global estimation space, but in spite of this shortcoming it is a thoughtful contribution which can serve to generate more reflections. In this light, there are a few areas where the paper could benefit from more comprehensive considerations:

**Scope of GBD**

 The GBD initially was intended to provide a comprehensive analysis of health loss. The key outputs were mortality by cause, DALYs and healthy life expectancies (HALE). The latter measure is not mentioned in the paper but is probably the most suitable single measure for assessment of progress towards the health goal of the sustainable development goals (health and wellbeing for all).

 In recent years, the GBD study has become synonymous with any estimate of individual disease burden, from maternal mortality to mental illness, and is an essential component of indexes such as those for IHME health SDG and the IHME quality and access index ^1^^,^^2^. WHO (as well as UNICEF and UNAIDS) has traditionally generated estimates through its programs, working with expert groups with broad representation from the world’s leading scientists in their respective fields. WHO fitted these disease-specific estimates into an overall envelope of the total number of deaths in the world to produce an overall picture of the burden of disease and ensure that overestimation would not occur. WHO’s decision to rely much more on IHME’s GBD for the overall burden of disease process makes sense. A key point is however the extent to which the specific WHO programs will be able to continue to produce independent estimates of disease burden in their respective areas, through their own expert group mechanisms, with IHME involvement where appropriate, and supported by solid investments by WHO.

**Global actors**

 The focus of the paper is on WHO and the World Bank. There are however other actors that need to be considered when moving forward. For instance, the United Nations Population Division produces estimates of the current and future population for each country on a regular basis. These estimates are produced in collaboration with countries. IHME is now using its own population estimates and this has a major impact on all GBD estimates.

 A very different actor in global health is the Lancet. The journal is mentioned frequently in the paper but its role is not examined. The Lancet has been instrumental in driving the global health agenda during the past decades and has been an effective vehicle for the publication of UN health estimates. The Lancet has been publishing volumes on the GBD with dazzling frequency and details on country statistics and rankings. The Lancet is owned by Elsevier, one of the largest publishing companies in the world, with a different set of institutional ethics and motivations that are worth considering in the debate.

**Monitoring**

 There is a tendency to increasingly use the GBD for the monitoring of progress in the context of major international health goals, most notably the SDG. The GBD study can produce almost any index to assess country progress for any year through the modelling exercises ^1^. The distinction between actual country progress and prediction becomes blurred ^3^. This is not a good practice and certainly puts countries in the role of spectators.

**Countries**

 The paper rightly raises concerns on the role of countries in the GBD exercise. This is not unique to the IHME GBD and is also a challenge for WHO and other UN agencies. WHO’s consultation processes to share estimates with countries for inputs (e.g. bring in new data, challenge methods and assumptions) are important but need considerable strengthening to make countries a full partner in these processes. Until today, IHME has invested far too little in making tools available to countries. In spite of all its sophistication it should be possible to develop GBD tools that can be put in the hands of countries and with default values and methods regularly updated through a web-based process. This would be a major step forward, especially if combined with analyses to identify data gaps and what can be done to reduce those gaps. The SDG have clearly laid out an agenda where countries are much more central than global actors. This deserves a systematic approach by WHO and its partners including IHME.

I confirm that I have read this submission and believe that I have an appropriate level of expertise to confirm that it is of an acceptable scientific standard.

## References

[1] : Population and fertility by age and sex for 195 countries and territories, 1950-2017: a systematic analysis for the Global Burden of Disease Study 2017. *The Lancet*.392(10159) : 10.1016/S0140-6736(18)32278-5 1995-2051 10.1016/S0140-6736(18)32278-5 PMC622791530496106

[2] : Measuring progress from 1990 to 2017 and projecting attainment to 2030 of the health-related Sustainable Development Goals for 195 countries and territories: a systematic analysis for the Global Burden of Disease Study 2017. *The Lancet*.392(10159) : 10.1016/S0140-6736(18)32281-5 2091-2138 10.1016/S0140-6736(18)32281-5 PMC622791130496107

[3] : Monitoring country progress and achievements by making global predictions: is the tail wagging the dog?. *The Lancet*.2018;392(10147) : 10.1016/S0140-6736(18)30586-5 607-609 10.1016/S0140-6736(18)30586-5 29661480

# Response

**Published:** 2020-01-29

**Authors:** Marlee Tichenor

Thank you for your careful analysis of our article and for your very important critiques and references, and we have attempted to address your most important points. On top of the expansions and corrections we mention in the main response text, we have also included a brief explanation of healthy life expectancy (HALE).

# Peer review: Reviewer response for version 1

**DOI:** 10.21956/wellcomeopenres.16376.r34902

**Published:** 2019-03-04

**Authors:** Carla AbouZahr

**Affiliations:** 1 Bloomberg Data for Health Initiative, Melbourne, Australia; 2 CAZ Consulting Sárl, Geneva, Switzerland

This is a timely and welcome effort to trace the history of the origins and evolution of the GBD. The article covers two interrelated topics: the evolution of the GBD since its origins in the 1993 World Development report; and the implications of the recently signed MOU between IHME and WHO, described by the editor of the Lancet as ending “ a cold war” between the two institutions. The authors present a summary of the development of GBD and raise important questions about the role of GBD estimates with regard to global health governance, health policy and relationships between donors and countries. However, there are some gaps in the historical account and in the analysis that should be addressed prior to publication.  

 The *Methods *section describes the two sources upon which the paper is based: published articles on GBD, along with “media representations on WHO, IHME and World Bank partnerships” and notes taken during public events, two of which were IHME organised, including the 20 ^th^year anniversary. I do not think this is a sufficient basis upon which to draw conclusions. It would have been advisable to have consulted with other individuals and institutions involved in the evolution of GBD.  

 The reporting of the 1993 World Development Report appears to have been largely influenced by reference 20, the 2017 paper by Murray and Lopez in which three individuals are singled out as contributors to WDR 1993 (with a footnote to “many others whose labour was essential”). There is no mention of the large teams drawn from multiple agencies, including not only the World Bank and WHO but also academia and other UN agencies. This is a weakness because the institutional support provided by global agencies was instrumental for the long-term sustainability of GBD.  

 The authors quote the original purpose of GBD, to provide “a detailed study of health loss to provide the basis for objective assessments about the probable benefits of applying packages of interventions.” This is correct, but the drive to “decouple epidemiological assessment …. from advocacy by interest groups of particular health policies or interventions,” seems to have crept in later, along with criticism of the estimation methods used by WHO, UNICEF and others ^1^.

 The authors might have taken a more critical view of this judgement and questioned whether technical experts working on specific health topics are incapable of “a neutral, objective perspective” (page 4). I wonder if they discussed these issues with WHO staff. There is no mention of the various technical evaluation reference groups established by WHO to guide the estimation process and provide objective, independent advice on the methods and results. See for example, http://cherg.org/main.html and http://www.epidem.org. 

 Given that this paper purports to trace the evolution of GBD they could have paid more attention to the ways in which the GBD evolved to take account of the criticisms that arose from the beginning. The authors state that age weighting was dropped in the 1997 GBD but this should be checked. I believe that age and time discounting were not dropped until the 2010 GBD following the advice from an array of scientists and ethicists ^2^. The authors affirm that “the architects of the GBD have carefully reassessed ….. the disability weights, ….. among other changes over the decades” but provide no specifics. 

 They might also have considered whether other criticisms of GBD have been sufficiently addressed, for example, neglect of the gender dimensions of health ^3^, inadequate examination of the underlying social and economic determinants of health ^4^; and the exclusion of certain adverse health outcomes, notably stillbirths ^5^^,^^6^. More discussion of the ways in which the DALY metric has been modified (or not) over time and the implications of such changes would have added value to the history ^7^. 

 The authors could have been more inquisitive about how the data needed to fuel the annual GBD updates are gathered and from whom. Perhaps part of the rationale for the MOU is to enable IHME to benefit from WHO’s privileged access to country data? They might also have examined the unintended effects of the annual GBD revisions which rely on ever more complex and data-hungry algorithms. The risk is that the GBD turns into an academic extractive industry that takes valuable resources – data – from countries and adds value in a faraway institution that is accountable only to its own funding partner.  

 An aspect that the authors do not mention is that GBD undertaking would not be possible without the huge advances in information technology behind the complex calculations and algorithms that drive the GBD. Such IT capacities and skills are not widely available, even in many middle-income settings, let alone in the global south. In their absence, it is impossible to truly understand the inner workings of the GBD or to replicate what it does.  

 The section on users and uses of GBD is very interesting but could have been enriched by a more careful analysis of the ways in which GBD is used and by whom. Most users welcome the GBD for its overview of global patterns of death and disease and value IHME innovation in areas such as health futures, trend forecasting, costing and cost-effectiveness, and visualization of complex data. However, the GBD work is contentious not only because estimates differ between WHO and IHME but also because increasingly the indicator estimates are used for monitoring progress on indicators, such as those included in the SDGs, that are highly politicised. Using the estimates for monitoring is technically problematic; in practice the estimates are predictions of indicator values for a given year extrapolated from past trends. The use of predictions for monitoring purposes has been criticised by Murray among others ^8^^,^^9^.

 In discussing the available information on users, the authors could have thought more about the reasons for the relative lack of use by country decision makers. Did the authors consult with some of the country policy makers whose data are used in the development of GBD? The GBD annual updates typically involve a complete re-estimation of the whole time series rather than simply adding new values for recent years, effectively shifting the goal posts at each reiteration of the estimates. From a policy-maker’s perspective in a particular country, it is hard to decipher whether the updates reflect new data or changes in the estimation methods. This can seem highly confusing, not to say dubious, even in countries with strong statistical systems ^10^. The use of confidence intervals to reduce the impact of the differences may allay the concerns of technical experts but can be hard to explain to policy makers.

 The authors do not suggest how the GBD might be made more useful and relevant to country policy makers or how to reduce the inevitable tensions arising when global estimates differ from country values. They could draw on the WHO experience of sharing its estimates with countries prior to publication and working with them to explain why the modelled estimates differ from country reported values. Inevitably there are disagreements, but the approach has contributed to improved understanding of the estimation process, capacity development and trust building between countries and agencies. IHME does not engage in similar country consultation processes prior to publication.

 I agree with the author’s concerns about the implications for global health governance and health policy of “an alliance of global burden of disease estimates” which may potentially create a “global health data monopoly.” They might have explored  this issue in more detail. While this is not the first attempt to improve collaboration between WHO and IHME, the tenor in this instance seems to place IHME, with its vast human and financial resources, firmly in the lead, rather than on equal terms with WHO. The authors suggest that the alliance will require WHO to “reconcile estimates and methodologies from IHME with its own estimates and country reported numbers” and that the GBD findings would be published in the Lancet, “before being used in official WHO documents”. If this is correct, WHO will find itself in the position of having to justify IHME estimates to its own Member States! 

 It would have been instructive had the authors pursued some of their contacts to answer some more detailed questions. What is the balance of gains and losses if global and country health policy-makers have access to only a single source of global health data? What are the implications for WHO’s governance architecture if the secretariat has to take on the responsibility of reconciling country data with IHME estimates? Will WHO continue to develop its own estimates? What will happen to the technical advisory mechanisms established by WHO programmes that offer good models of inter-agency collaboration? Are there potential conflicts of interest given the growing role of the Gates Foundation in both IHME and WHO as well as disease-focussed health programmes around the world? 

 The authors’ overall conclusions seem incontrovertible. The future development of the GBD has major implications for countries and for the international development system in terms of governance, health policy, transparency, ownership, and accountability. Further open discussion of these issues is much needed.  

 Editorial comments:

- Table 1 should include the WHO update on methods on 2018 ( https://www.who.int/healthinfo/global_burden_disease/GlobalDALY_method_2000_2016.pdf
- Table 1 should provide correct citations.
- Table 1 Line 1 there is no mention of WHO institutional support to the 1993 WDR.
- Table 1 Line 3 There were several WHRs that were based on GBD analyses and these should be cited correctly, 1999, 2000, 2001, 2002. All used the concept of DALYs.   
- Table 12 The comments “new methodologies for finding mortality” should be explained.
- Table 1 Lines 3 and 4 Correct citation of the World Health Reports does not include author names; in any case correct name would be Mafat.
- Page 3 The reference to the 1993 World Development Report is missing. 
- Footnote page 3 should read ‘ as three among’.

I confirm that I have read this submission and believe that I have an appropriate level of expertise to confirm that it is of an acceptable scientific standard, however I have significant reservations, as outlined above.

## References

[1] : Monitoring global health: time for new solutions. *BMJ*.2004;329(7474) : 10.1136/bmj.329.7474.1096 1096-100 10.1136/bmj.329.7474.1096 15528624PMC526127

[2] : WHO methods and data sources for global burden of disease estimates 2000-2016. *Department of Information, Evidence and Research, WHO, Geneva*.2018;

[3] : Gender Differentials in Health.2006; 21250310

[4] : The social determinants of health: coming of age. *Annu Rev Public Health*.2011;32: 10.1146/annurev-publhealth-031210-101218 381-98 10.1146/annurev-publhealth-031210-101218 21091195

[5] : Valuing Stillbirths. *Bioethics*.2015;29(6) : 10.1111/bioe.12120 413-23 10.1111/bioe.12120 25395144PMC4706157

[6] : WHO publishes new estimates on congenital syphilis. *[accessed 1st March 2019]*. Reference source

[7] : The evolution of the disability-adjusted life year (DALY). *Socio-Economic Planning Sciences*.2015;49: 10.1016/j.seps.2014.12.002 10-15 10.1016/j.seps.2014.12.002

[8] : Towards good practice for health statistics: lessons from the Millennium Development Goal health indicators. *The Lancet*.2007;369(9564) : 10.1016/S0140-6736(07)60415-2 862-873 10.1016/S0140-6736(07)60415-2 PMC713786817350457

[9] : Monitoring country progress and achievements by making global predictions: is the tail wagging the dog?. *The Lancet*.2018;392(10147) : 10.1016/S0140-6736(18)30586-5 607-609 10.1016/S0140-6736(18)30586-5 29661480

[10] : Credibility in published data sources. *The Lancet*.2019;393(10168) : 10.1016/S0140-6736(18)32844-7 225-226 10.1016/S0140-6736(18)32844-7 30663588

# Response

**Published:** 2020-01-29

**Authors:** Marlee Tichenor

Thank you for your careful analysis of our article and for your very important critiques and references, and we have attempted to address your most important points. We have incorporated the 2018 “WHO methods and data sources” technical paper as you suggested, being particularly careful about highlighting the evolution of and debate around disability weights. As we mention in the new version of the article, we highlight the conflict of interest at the heart of BMGF funding the (potentially) primary mode of measuring its own success in pushing its health investments in the SDGs. Thank you for emphasising this point. We have also addressed your editorial comments, and we have been more careful about the limitations of the research on which this article was based.

# Peer review: Reviewer response for version 1

**DOI:** 10.21956/wellcomeopenres.16376.r34901

**Published:** 2019-03-04

**Authors:** Peter Byass

**Affiliations:** 1 Department of Epidemiology and Global Health, Umeå University, Umeå, Sweden

**Introduction:**

 There is a danger of over-personalising the admittedly somewhat fraught history of the Global Burden of DIsease (GBD) programme over a quarter of a century. The consistent personal aspect of this history is tied up around Chris Murray and Alan Lopez, who at the outset of the process were WHO staffers - a point that does not come across clearly from the start here. Not surprisingly, they have moved institutions and attracted different funding support several times over the whole period. Thus WHO and the World Bank were involved from the start, and continue to be so; the 2018 MoU between WHO and GBD is the latest development in this long saga. There have also been significant collaborations along the way, for example the development of the GATHER guidelines for reporting global health estimates ^1^, which was the direct product of technical work involving both WHO and IHME, and should be mentioned here. However, there have certainly been institutional and personal frictions associated with the complex history of GBD, which are important to explore and understand.

**The 25 years of GBD:**

 This section describes much of the practice and politics of GBD development, but in parts has a more journalistic than academic style, and particularly **lacks a conceptual framework** as the basis for understanding some of the successes and failures along the way. My suggestion would be that insights into this process should be based on understanding and comparing the varying world views of the key institutions and individuals involved. In a 2010 commentary ^2^, when WHO-GBD relationships were particularly difficult, I suggested that an important source of difference in understanding arose between the UN world view, in which member states are consitutents who need to be carried along in the process of deriving estimates, versus academic institutions' world view in which research sources and methods need to processed as cleverly as possible and promulgated to the world.

 (note two typos here in the acronym for Bill and Melinda Gates Foundation, should be BMGF)

**On the production of GBD:**

 Partly deriving from GBD's distinctive world view, of seeking to assert estimates of everything for everywhere at all times - which is a noble academic ambition - a lot of gap-filling has to be done because the ideal underlying data simply don't exist. In many instances, imputed estimates turn out very plausibly, and therefore, it might be argued, usefully. However, taking this approach does have its dangers, and there will be examples where for some reason the estimating process falls over (particularly if over-sophisticated modelling is applied to very sparse data). An interesting example was seen in GBD estimates of dengue deaths and case numbers, which, when divided to derive case-fatality rates, improbably showed that the USA had the highest dengue case-fatality rate in the world ^3^. Thus there seems to be a case for more effective plausibility checking mechanisms for GBD estimates, rather than necessarily considering what comes out of the modelling as some kind of "truth".

**Who uses these estimates?:**

 Perhaps a more important question is "How are these estimates used?". While the point is made that BMGF mandates and supports the GBD process as a means of prioritising its global health priorities, you should also discuss this point as a potential conflict of interest. Every development agency rightly delights in their own successes, but this becomes tricky if they are also supporting the underlying metrics. At the same time BMGF is the largest non-government funder of WHO!

I confirm that I have read this submission and believe that I have an appropriate level of expertise to confirm that it is of an acceptable scientific standard, however I have significant reservations, as outlined above.

## References

[1] : Correction: Guidelines for Accurate and Transparent Health Estimates Reporting: the GATHER statement. *PLOS Medicine*.2016;13(8) : 10.1371/journal.pmed.1002116 10.1371/journal.pmed.1002116 PMC497840827504831

[2] : The imperfect world of global health estimates. *PLoS Med*.2010;7(11) : 10.1371/journal.pmed.1001006 e1001006 10.1371/journal.pmed.1001006 21152416PMC2994666

[3] : The elusive global burden of dengue. *The Lancet Infectious Diseases*.2016;16(6) : 10.1016/S1473-3099(16)00076-1 629-631 10.1016/S1473-3099(16)00076-1 26874620

# Response

**Published:** 2020-01-29

**Authors:** Marlee Tichenor

Thank you for your careful analysis of our article and for your very important critiques and references, and we have attempted to address your most important points. As we mentioned in the main response text, we have included a framework for conceptualising the different modes of global health estimate production, building from your own conceptual framework that compares the “UN world view” with that of academic institutions (Byass 2010). As we mention in a footnote in the article, we are grateful for your emphasis on BMGF's conflict of interest with regards to funding both global health investments and the potentially primary mode of measuring their success in the SDG framework.
